# The Contributions of the Cerebellar Peduncles and the Frontal Aslant Tract in Mediating Speech Fluency

**DOI:** 10.1162/nol_a_00098

**Published:** 2024-08-15

**Authors:** Sivan Jossinger, Maya Yablonski, Ofer Amir, Michal Ben-Shachar

**Affiliations:** The Gonda Multidisciplinary Brain Research Center, Bar-Ilan University, Ramat-Gan, Israel; Department of Communication Disorders, Sackler Faculty of Medicine, Tel-Aviv University, Tel-Aviv, Israel; The Department of English Literature and Linguistics, Bar-Ilan University, Ramat-Gan, Israel

**Keywords:** cerebellum, DTI, probabilistic tractography, speaking rate, speech production, white matter

## Abstract

Fluent speech production is a complex task that spans multiple processes, from conceptual framing and lexical access, through phonological encoding, to articulatory control. For the most part, imaging studies portraying the neural correlates of speech fluency tend to examine clinical populations sustaining speech impairments and focus on either lexical access or articulatory control, but not both. Here, we evaluated the contribution of the cerebellar peduncles to speech fluency by measuring the different components of the process in a sample of 45 neurotypical adults. Participants underwent an unstructured interview to assess their natural speaking rate and articulation rate, and completed timed semantic and phonemic fluency tasks to assess their verbal fluency. Diffusion magnetic resonance imaging with probabilistic tractography was used to segment the bilateral cerebellar peduncles (CPs) and frontal aslant tract (FAT), previously associated with speech production in clinical populations. Our results demonstrate distinct patterns of white matter associations with different fluency components. Specifically, verbal fluency is associated with the right superior CP, whereas speaking rate is associated with the right middle CP and bilateral FAT. No association is found with articulation rate in these pathways, in contrast to previous findings in persons who stutter. Our findings support the contribution of the cerebellum to aspects of speech production that go beyond articulatory control, such as lexical access, pragmatic or syntactic generation. Further, we demonstrate that distinct cerebellar pathways dissociate different components of speech fluency in neurotypical speakers.

## INTRODUCTION

Humans produce about 16,000 words every day, at an astounding speed of more than 150 words per minute (Amir, 2016; Mehl et al., 2007; Rodero, 2012). Such fast and fluent speech production depends upon complex interactions between motor, sensory, and cognitive systems underpinning different aspects of speech production, such as articulatory control, phonological encoding, conceptual framing, and lexical access (Hickok, 2012). Despite these interactions, most studies investigating speech fluency focus on either articulatory control or lexical access, but not both. Here, we combine the two perspectives to allow a comprehensive understanding of the neural pathways associated with fluent speech production in neurotypical adults.

Lexical access, including lexical search, selection, and retrieval, is typically measured using verbal fluency tasks. In these tasks, participants are asked to produce as many words as possible within 60 s under a specific criterion: words beginning with a certain letter (i.e., phonemic fluency) or a semantic category (i.e., semantic fluency). Verbal fluency is often assessed in clinical populations such as persons with aphasia (Bose et al., 2022), dementia (Libon et al., 2009), multiple sclerosis (Blecher et al., 2019), Parkinson’s disease (Henry & Crawford, 2004), and more. Importantly, performance in the verbal fluency task is influenced by adequate word selection and avoidance of repetition. Therefore, these tasks are used to assess not only lexical knowledge, but also executive functions such as working memory and inhibition (Amunts et al., 2020; Shao et al., 2014). Indeed, verbal fluency is a central task in neuropsychological batteries that assess executive functions (Kramer et al., 2014; Shao et al., 2014).

Fluent speech production is also quantified using measures of speech rate. In contrast to the timed and highly constrained nature of verbal fluency tasks, speech rate measures, such as speaking rate and articulation rate, are based on a relatively naturalistic setting of spontaneous speech samples. Broadly, speech rate is calculated as the number of spoken units (syllables or words) produced within a time unit (second or minute). Speaking rate is measured across continuous segments of speech, which may include pauses, repetitions, and revisions. Thus, it is considered a global measure of verbal output and language proficiency (Costello & Ingham, 1984; Howell et al., 1999). Articulation rate, on the other hand, is based only on fluent utterances after excluding any kind of disfluency and thus considered a measure of articulatory motor control (Walker et al., 1992). Generally, speech rate (as quantified by both measures) affects the speakers’ intelligibility, fluency, and communication efficiency (Amir, 2016; Sturm & Seery, 2007). Measures of speech rate are typically used in the clinical assessment of various speech disorders, such as apraxia of speech (Kent & Rosenbek, 1983), dysarthria (Kent et al., 1987), and persistent developmental stuttering (Andrade et al., 2003).

Although speech fluency involves both lexical access and articulatory control, studies in the field tend to focus either on linguistic aspects of speech or on motor aspects of speech, but not both. In an attempt to bridge this gap, Hickok has recently suggested the hierarchical state feedback control model of speech production (Hickok, 2012). This model synthesizes between psycholinguistic and motor control approaches and suggests an integrated brain circuitry of speech production. The current understanding of the brain circuits that control fluent speech production, however, stems largely from studies in patients. Hence, in the current study we wish to evaluate the different interpretations of speech fluency and evaluate the neural substrates that stand at the base of fluent speech production in neurotypical adults.

According to computational models of speech production, the cerebellum is a key node in different aspects of fluent speech production (Hickok, 2012; Tourville & Guenther, 2011). Indeed, patients with cerebellar lesions exhibit impaired verbal fluency abilities and significantly slower speaking rate and articulation rate compared to controls (Ackermann et al., 1992; Peterburs et al., 2010). Neuroimaging data point to a significant cerebellar activation during both verbal fluency tasks (Halari et al., 2006; Schlösser et al., 1998) and tasks that involve change in articulation rate (Riecker et al., 2005, 2006). Naturally, the involvement of the cerebellum in verbal fluency tasks could stem from the fact that fluency tasks use the articulatory system. Therefore, to make a functional segregation, the contribution of the cerebellum to each of these measures should be evaluated in the same participants.

Apart from the cerebellum, neural control of speech recruits a distributed cortical network (Hickok, 2012; Tourville & Guenther, 2011). The cortex and the cerebellum are structurally connected via long range white matter pathways which enable efficient communication of signals across considerable distance. As the exclusive bridge between the cerebellum and extra-cerebellar regions, the structural properties and organization of the cerebellar peduncles are important for understanding the neural basis of fluent speech production.

Cerebellar input and output information is carried by three major white matter pathways known as the cerebellar peduncles (CPs): the inferior cerebellar peduncle (ICP), the middle cerebellar peduncle (MCP), and the superior cerebellar peduncle (SCP). The ICP is a cerebellar input pathway, feeding signals from the inferior olive and spinal cord into the cerebellar cortex (Perrini et al., 2013). The ICP was shown to be implicated in articulation rate among people with developmental stuttering. Developmental stuttering was previously associated with abnormalities in the microstructural properties of the bilateral ICP (Connally et al., 2014; but see Jossinger et al., 2021, 2022, for contradicting results). A recent study in young children who stutter showed that microstructural differences in the ICP emerge early in development (Johnson et al., 2022). In adults with developmental stuttering, but not in neurotypical speakers, the microstructural properties of the ICP was shown to be correlated with articulation rate (Jossinger et al., 2021; Kronfeld-Duenias et al., 2016).

The SCP is a major output pathway transmitting signals from the cerebellum into the contralateral cerebral cortex via the thalamus. The MCP is a major input pathway feeding signals from the cerebral cortex into the contralateral cerebellar cortex, decussating at the level of the pontine nucleus. Together, the SCP and MCP form the cerebro-cerebellar loop which allows transferring information from the cerebellum to the cerebral cortex and vice versa. Computational models of speech production hypothesize that the cerebro-cerebellar projections contribute to the feedforward control of speech by mapping between the desired speech sound and its appropriate articulatory gesture (Tourville & Guenther, 2011). Recent data show that the cerebro-cerebellar connectivity is also associated with speech-related cognitive demands, such as verbal working memory (Sobczak-Edmans et al., 2019) and story comprehension (Castellazzi et al., 2018). Functional magnetic resonance imaging (fMRI) studies have shown that verbal fluency tasks cause a significant activation in the cerebellum, together with prefrontal and temporo-parietal areas (Gurd et al., 2002; Hubrich-Ungureanu et al., 2002; Schlösser et al., 1998). On this basis, cerebellar activity during verbal communication may reflect not only motor aspects of speech production, but also cognitive demands of word generation.

The potential involvement of the cerebro-cerebellar loop in the fluent production of speech is further supported by the anatomical connections found between the cerebellum and the prefrontal cortex (Kelly & Strick, 2003; Middleton & Strick, 1994; Palesi et al., 2017). Within the prefrontal cortex, feedforward control of speech involves the activation of the inferior frontal gyrus (IFG) and the supplementary motor area (SMA; Hickok, 2012; Tourville & Guenther, 2011). The IFG and SMA were recently shown to be connected via the frontal aslant tract (FAT; Catani et al., 2012). To date, the involvement of the FAT in speech fluency was mainly studied in clinical populations. For example, Kronfeld-Duenias et al. (2016) showed that adults who stutter demonstrate abnormal microstructure within the FAT compared to fluent speakers. This structural difference was also accompanied by a correlation between the microstructural properties of the FAT and articulation rate, association which was not evident in fluent speakers. Interestingly, in patients with multiple sclerosis and in patients with chronic aphasia, the FAT was associated with verbal fluency measures (Blecher et al., 2019; Catani et al., 2013; Li et al., 2017).

In the current study, we evaluated the contribution of the CPs and the FAT to the different measures of speech fluency in neurotypical adults. Participants (*N* = 45) underwent an unstructured interview to assess their natural speaking rate and articulation rate, and completed timed semantic and phonemic fluency tasks to assess their verbal fluency. Diffusion MRI data were measured in the same group of participants using a single-shell high angular resolution imaging sequence (Tuch et al., 2003). The automatic fiber segmentation and quantification package (AFQ; Yeatman et al., 2012) was adapted in order to delineate the bilateral SCP and MCP as they decussate at the level of the inferior colliculi and pons, respectively. Microstructural properties were extracted from the CPs and the FAT, and entered into correlation analyses with measures of speech fluency. Based on computational models of speech production (Hickok, 2012; Tourville & Guenther, 2011), and on previous neuroimaging reports (Castellazzi et al., 2018; Gurd et al., 2002; Hubrich-Ungureanu et al., 2002; Schlösser et al., 1998; Sobczak-Edmans et al., 2019), we expected that the microstructural properties of the cerebro-cerebellar loop (i.e., SCP and MCP) would be correlated with both articulatory and verbal aspects of speech production. Importantly, this analysis is still exploratory, due to the lack of prior data or modeling work relating each CP to specific language functionalities. Based on previous studies of structural connectivity in clinical populations (Blecher et al., 2019; Catani et al., 2013; Li et al., 2017), we hypothesized that the microstructural properties of the FAT would be associated with verbal fluency measures in the current sample of neurotypical adults. Lastly, based on our previous findings (Jossinger et al., 2021; Kronfeld-Duenias et al., 2016), neurotypical adults were not expected to show an association between articulation rate and the ICP or the FAT.

## MATERIALS AND METHODS

### Participants

Forty-five neurotypical adults (29 females; mean age 26.45 ± 3.72 years; Table 1) were recruited for this study. All the analyses reported here are completely new, but some data from this sample have been reported as part of a research project focusing on associations between well-known language pathways and word structure (Yablonski & Ben-Shachar, 2020; Yablonski et al., 2021). All participants were right handed as estimated by the Edinburgh handedness inventory (Oldfield, 1971; Table 1), and had no history of a diagnosed speech impairment, learning disability, or neurological condition. All participants were native Hebrew speakers who speak and read English as L2. One participant was referred to neurological follow-up due to an incidental finding, but otherwise had normal anatomical structure and diffusion values and was thus not excluded from analysis. Participants were paid 200 NIS for their participation. All participants signed a written informed consent before participating in the study. This study was approved by the Helsinki committee of the Sheba Medical Center, by the Institutional Review Board of Tel Aviv University, and by the ethics committee of the Faculty of Humanities in Bar-Ilan University.

**Table 1.** Sample characteristics (*N* = 45).

	Mean	*SD*	Range
Demographics			
Gender	16M/29F	–	–
Age (years)	26.45	3.72	[20.23, 34.87]
Education (years)	14.56	2.13	[12, 20]
Handedness	96.89	5.90	[80,100]

Speech rate			
Speaking rate (syllables/s)	5.04	0.68	[3.49, 6.78]
Articulation rate (syllables/s)	6.42	1.05	[4.57, 9.08]

Verbal fluency			
Phonemic fluency (Words/min)	43.78	9.83	[17, 68]
Semantic fluency (Words/min)	66.96	12.22	[46, 99]

### Speech Fluency Assessment

#### Speaking task

Speaking rate and articulation rate were measured over audio recordings of an unstructured interview. The interview took place in a quiet room and was simultaneously recorded with a Sennheiser PC21-II noise-canceling microphone (Sennheiser, 2023) and with a Sony HDR-CX405 digital video camera (Sony, 2022). Audio signals from the microphone were digitally recorded using Audacity (Audacity Team, 2021) on a mono channel with a sampling rate of 48 kHz (16 bit). The participant was asked to talk about a neutral topic (e.g., a recent travel experience, a movie, a book), for about 10 min. The experimenter (M.Y.) refrained from interrupting, asking open questions only when the participant was having difficulty finding a topic to talk about.

#### Speech rate measures

Two speech rate measures were calculated: Speaking rate and articulation rate. Both measures were calculated over the audio recordings of the unstructured interview. Articulation rate was calculated as the ratio between the total number of analyzed syllables and the time it took the participant to produce them (i.e., syllables/s), after excluding disfluent utterances (Ambrose & Yairi, 1999; Amir, 2016; Amir & Grinfeld, 2011; Rochman & Amir, 2013). Speaking rate was calculated in the same way, on the entire speech segment without exclusions. For this purpose, two trained research assistants first transcribed each interview and annotated any disfluent epochs (mostly naturally occurring disfluencies, such as hesitations, repetitions, and revisions). An utterance was defined based on three criteria: (1) communicated an idea, (2) had a well-defined intonation contour, and (3) was grammatically complete. Overall, a fixed number of 50 utterances were used to calculate speaking rate, and 12.8 ± 6.36 utterances were used to calculate articulation rate.

#### Verbal fluency tasks

Normed Hebrew versions of the phonemic and semantic fluency tasks were implemented (Kavé, 2006; Kavé & Knafo-Noam, 2015). Participants were asked to produce as many words as possible within 1 min according to a criterion: words beginning with a certain letter (phonemic fluency tasks) or a semantic category (sematic fluency tasks). Each task was repeated three times with different criteria. For the Phonemic fluency task, participants were asked to produce words that begin with the letters Bet (/b/), Gimel (/g/), and Shin (/ʃ/ or /s/). For the semantic fluency task, participants were asked to produce words that belong to the categories animals, fruits and vegetables, and vehicles. The administration order of the tasks and the different items within each task was kept constant across participants, starting with the phonemic fluency task and followed by the semantic fluency task. The experimenter (M.Y.) read each criterion aloud, and participants’ oral responses were recorded and transcribed offline.

#### Verbal fluency measures

First, responses in both tasks were screened according to the guidelines described in Kavé and Knafo-Noam (2015). Accordingly, repetitions and erroneous responses were removed. For example, in the semantic fluency task, names of subcategories (e.g., birds) were not counted if the participant also produced specific exemplars within the subcategory (e.g., raven, pigeon). The total number of correct unique responses was coded per criterion and summed across the three criteria within each task. We also calculated a standardized verbal fluency score based on age-appropriate Hebrew norms (Kavé & Knafo-Noam, 2015). The analysis of standardized verbal fluency scores in the current sample is reported in a previous paper (Yablonski et al., 2021). Note, however, that this previous paper did not examine the associations between verbal fluency measures and the CPs, which is the focus of the current study.

### Behavioral Intercorrelation Analysis

Correlations between speech fluency components were calculated using the open-source R environment for statistical analysis (R Core Team, 2013). Specifically, we calculated Spearman’s correlations between speaking rate, articulation rate, phonemic fluency, and semantic fluency, resulting in an intercorrelation matrix of six correlations overall. To account for multiple comparisons, we controlled the false discovery rate (FDR) at a level of 5% (Benjamini & Hochberg, 1995).

### White Matter Analysis

#### MRI data acquisition

MRI scans were conducted on a 3T Siemens Magnetom Prisma scanner (Siemens Medical Solutions, 2022) at the Strauss Center for Computational Neuroimaging at Tel Aviv University, with a 64-channel head coil. The MRI protocol included standard anatomical and diffusion imaging sequences, as detailed below. Functional MRI scans were also included in the scanning protocol but are not reported here.

##### T1 image acquisition.

High-resolution T1-weighted anatomical images were acquired using a magnetization prepared rapid acquisition gradient echo (MPRAGE) protocol (repetition time [TR] = 2.53 s, echo time [TE] = 2.99 ms, flip angle = 7°, 1 mm thick slices, matrix size: 224 × 224 × 176, voxel size: 1 × 1 × 1 mm).

##### Diffusion weighted image acquisition.

A standard diffusion MRI (dMRI) protocol was applied by means of a single-shot spin-echo diffusion-weighted echo-planar imaging sequence (86 axial slices, each 1.7 mm thick, no gap; field of view = 204 × 204 mm, image matrix size = 120 × 120 providing a cubic resolution of 1.7 × 1.7 × 1.7 mm, TR = 4,000 ms, TE = 58 ms). Sixty-four diffusion-weighted volumes (*b* = 1,000 s/mm^2^) and three reference volumes (*b* = 0 s/mm^2^) were acquired using a standard diffusion direction matrix. Multiband acceleration was used with slice acceleration factor of 2. Total acquisition time for the dMRI sequence was 4:48 min.

#### MRI data analysis

##### Software.

Data analysis was conducted using MATLAB 2012b (MathWorks, 2012). Data preprocessing was obtained using the open source mrDiffusion package (Vista Lab, 2022). Constrained spherical deconvolution (CSD) diffusion model was calculated using the mrTrix3 toolbox (https://www.mrtrix.org/; Tournier et al., 2019). Tract identification and quantification were executed with the automatic fiber segmentation and quantification (AFQ) toolkit (https://github.com/yeatmanlab/AFQ; Yeatman et al., 2012). Individual tracts were visually inspected using Quench, an interactive 3D visualization tool (https://web.stanford.edu/group/vista/cgi-bin/wiki/index.php/QUENCH; Akers, 2006).

##### Data preprocessing.

Preprocessing was implemented in the native space of each individual, following a published pipeline (Yablonski et al., 2021). This pipeline included several steps: (1) alignment of the T1 images to the anterior commissure–posterior commissure orientation; (2) motion- and eddy-current correction of DW-EPI data (Rohde et al., 2004); (3) registration of the diffusion weighted volumes to the averaged non-diffusion weighted volumes (b0); (4) registration of the mean b0 image to the T1 image using a rigid body mutual information maximization algorithm (implemented in SPM8; Wells et al., 1996); (5) application of the combined transform resulting from motion correction, eddy-current correction, and anatomical alignment to the raw diffusion data; (6) adjustment of the table of gradient directions to fit the resampled diffusion data (Leemans & Jones, 2009).

At the voxel level, the diffusion data were modeled twice, once with the tensor model and once with the constrained spherical deconvolution (CSD) model. Diffusion tensors were used to calculate, within each voxel, the commonly used diffusion metrics (e.g., fractional anisotropy), which were later used for the statistical analysis. Tensor modeling was carried out using “mrDiffusion.” Tensors were fit to the registered diffusion data using a robust least-square algorithm (RESTORE), which removes outliers at the tensor estimation step (Chang et al., 2005). Then, using tensor decomposition, we extracted the three eigenvalues and eigenvectors of the tensor, and calculated, within each voxel, fractional anisotropy (FA) and mean diffusivity (MD). Specifically, FA was calculated as the normalized standard deviation of the eigenvalues, and MD was calculated as the average of the three eigenvalues (Basser & Pierpaoli, 1996).

As the tensor model is notoriously known for its inability to account for multiple orientations within the same voxel (Jones et al., 2013), our individual tractograms were generated based on the CSD model. The CSD model estimates the fiber orientation distribution (FOD) within each voxel based on the spherical deconvolution between the diffusion weighted signal and an estimation of the diffusion response function (Tournier et al., 2004, 2007). To calculate the CSD model we used the “mrTrix” toolbox (Tournier et al., 2019). First, to estimate the response function we used the dhollander algorithm (implemented within the dwi2response function). This algorithm estimates the response functions for white matter, gray matter, and cortico-spinal fluid (CSF), separately, based on single-shell diffusion data. Next, to estimate the FOD, we applied constrained spherical deconvolution with up to eight spherical harmonics (lmax = 8) on the responses estimated within the white matter and the CSF. This step was carried out by the msmt_csd algorithm (implemented within the dwi2fod function; Jeurissen et al., 2014). Lastly, the FODs were used to generate individual tractograms using probabilistic tractography, as explained below.

##### Fiber tractography.

Individual tractograms were generated using probabilistic whole brain tractography, with the iFOD2 tracking algorithm (implemented in the mrTrix3 function tckgen).

As a first step, a whole brain white matter mask was generated from each participant’s structural T1 image. This procedure was done using the 5ttgen script, utilizing FSL (Jenkinson et al., 2012) tools to perform whole brain segmentation (Smith et al., 2004). The tracking was initialized from 500,000 random seeds within the white matter mask, and was restricted with the following parameters: (1) FOD amplitude threshold of 0.1; (2) 45° maximum angle between successive steps; (3) 0.85 mm step size; (4) streamline length between 50 mm to 200 mm. Streamlines that extended beyond the white matter mask were truncated. The resulting whole brain tractograms were then subjected to the automatic tract segmentation procedure.

##### Tract identification and segmentation.

**Figure 1.**
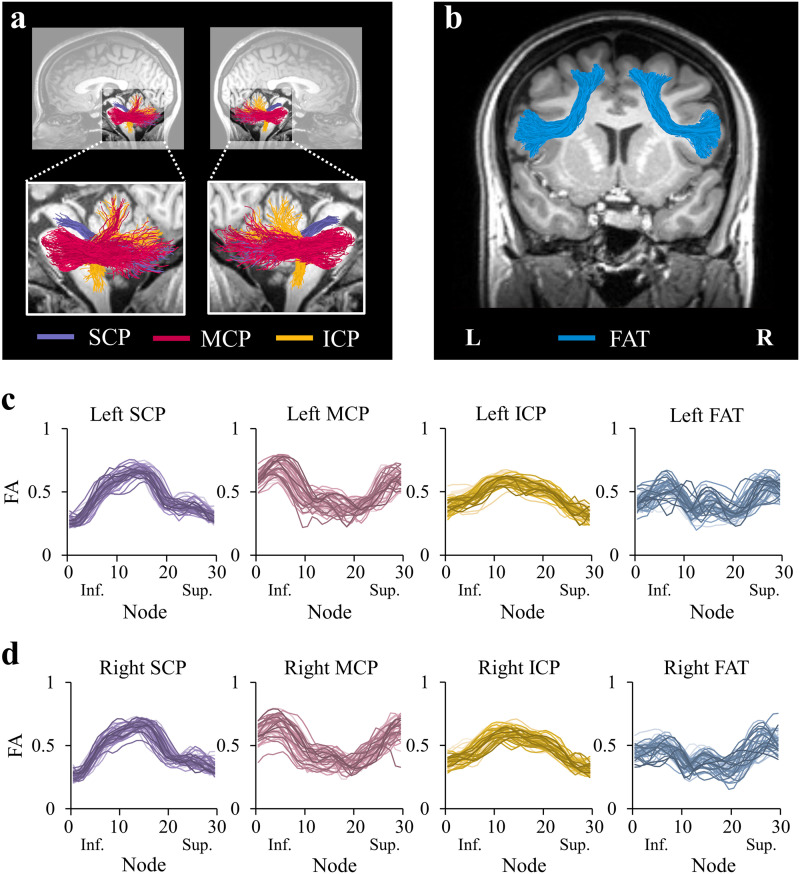
Tracts of interest. (A) Left and right cerebellar peduncles identified in a single participant (female, 34), overlaid on a T1 image. The cerebellar tracts identified are the SCP (purple), MCP (magenta), and the ICP (yellow). (B) Left and right FAT (blue) identified in a single participant (female, 24), overlaid on a T1 image. (C–D) Individual tract profiles show FA values at 30 equidistant nodes along the core of the left (C) and right (D) tracts of interest. Each subject is represented by a single line (*N* = 45). SCP = superior cerebellar peduncle; MCP = middle cerebellar peduncle; ICP = inferior cerebellar peduncle; FAT = frontal aslant tract; L = left; R = right; Inf. = inferior; Sup. = superior.

In each individual’s native space, we identified the bilateral CPs and the bilateral FAT (Figure 1). We targeted these tracts based on previous studies in clinical populations, directly linking the CPs (Figure 1A) and the FAT (Figure 1B) to speech fluency (Blecher et al., 2019; Catani et al., 2013; Connally et al., 2014; Jossinger et al., 2021; Kronfeld-Duenias et al., 2016; Li et al., 2017), and based on theoretical models of speech production (Hickok, 2012; Tourville & Guenther, 2011). Automatic segmentation of the tracts was carried out using AFQ. This method utilizes a multiple-region-of-interest (ROI) approach in which the tractograms are intersected with predefined ROIs using logical operations (Figure S1). In accordance with this method, the ROIs are universally defined on a template (Figure S1A), and then back-transformed to the participant’s native space using a nonlinear transformation (Figure S1B). The individual ROIs are intersected with the tractogram (Figure S1C) to isolate the tracts of interest (Figure S1D). To identify the CPs we propose a new protocol, which is based on several previous studies (Bruckert et al., 2019; Palesi et al., 2015, 2017). To identify the bilateral FAT, we used the protocol described in Kronfeld-Duenias et al. (2016), which is implemented in AFQ (publicly available at https://github.com/yeatmanlab/AFQ/tree/master/aslant).

Automatic segmentation of the CPs was recently introduced by Bruckert et al. (2019). This approach, however, delineates only the inferior parts of the SCP and MCP, before they decussate to the contralateral cerebral hemisphere. For this reason, the methods introduced in Bruckert et al. (2019) are better suited to delineate the CPs based on deterministic tractography, where fibers are less likely to decussate. On the other hand, probabilistic tractography approaches coupled with CSD modeling are more successful in following the CPs as they decussate. In order to segment the resulting tracts automatically, we revised the AFQ protocol including additional ROIs (see Figure S2 and Table S1, in the supporting information available at https://doi.org/10.1162/nol_a_00098, for detailed MNI coordinates of the ROIs).

The new set of ROIs used to identify the CPs were defined on the Montreal Neurological Institute (MNI) template (ICBM 2009a Nonlinear Asymmetric template; Fonov et al., 2011). For the SCP, a new ROI (SCP_superior_prob) was defined on an axial slice at the level of *z* = −10, encompassing a rectangle around the red nucleus (Oishi et al., 2009; Palesi et al., 2015). The SCP was then segmented using the newly defined ROI together with the previous SCP-ROIs described by Bruckert et al. (2019; Figure S2A). For the MCP, a new ROI (MCP_superior_prob) was defined as a parallelogram on an axial slice at the level of *z* = −16, including all the voxels occupied by the cerebral peduncle (Oishi et al., 2009; Palesi et al., 2017). The MCP was then segmented using the newly defined ROI together with the previous MCP-ROI introduced by Bruckert et al. (2019; Figure S2B). For the ICP, a new inferior ROI (ICP_inferior_prob) was defined to avoid fibers that enter the pons. The ICP was then segmented using the newly defined ROI together with the previous ICP-ROI introduced by Bruckert et al. (2019; Figure S2C).

The resulting tracts were cleaned automatically using a statistical outlier rejection algorithm implemented in AFQ. For the cleaning procedure of the SCP, MCP, and FAT, fibers were considered outliers if they were longer than 4 standard deviations from the mean fiber length and spatially deviated more than 4 standard deviations from the core of the tract. For the ICP, a relatively short tract, the length criterion was changed as previously done in Bruckert et al. (2019) such that fibers longer than 1 standard deviation from the mean fiber length were removed. The tracts are shown in Figure S3 in three representative subjects.

#### Brain-behavior correlation analysis

The analyses were restricted to the core segment of each tract, enclosed between the two waypoint-ROIs (Figure S2). This approach eliminates the extreme segments of the tracts which are highly variable between participants.

##### Mean-tract correlations.

Associations between diffusivity values and speech fluency measures were assessed using two-tailed Spearman’s rank-order correlations. As a first step, we calculated for each participant and each tract the average FA and average MD values across the core of the tract (i.e., tract FA and tract MD, respectively). Then, we assessed the simple correlations between tract FA and speech fluency measures. We controlled for multiple comparisons across eight tracts setting the FDR at a level of 5% (Benjamini & Hochberg, 1995). A similar analysis was conducted on tract MD values. Separate analyses were conducted for each behavioral measure (Xie et al., 2011).

##### Along-tract correlations.

It is well documented that diffusivity values vary significantly along the tract (Yeatman et al., 2011, 2012). This is also true for the CPs and the FAT, as shown in Figure 1C and D and Figure S4. We therefore applied a second analysis, assessing two-tailed Spearman’s correlations between speech fluency measures and diffusivity values at 30 equidistant locations along the core of the tract (Kruper et al., 2021). To account for multiple comparisons, significance was corrected using a nonparametric permutation test, controlling the familywise error (FWE) at a corrected alpha value of 0.05 (Nichols & Holmes, 2002). Clusters were considered significant if they satisfied two criteria: (1) each node within the cluster was significantly correlated with speech fluency at a level of alpha = 0.05 (uncorrected), and (2) the number of adjacent nodes composing the cluster should have been larger than a critical size, determined by the permutation algorithm (Nichols & Holmes, 2002; Yeatman et al., 2012). FWE correction was applied across the 30 nodes within each tract, with no further correction across tracts, behavioral measures, or diffusion metrics (for a similar approach, see, e.g., Yablonski et al., 2021; Yeatman et al., 2012).

##### Partial correlation.

To assess the specificity of associations and dissociate between tracts mediating lexical or articulatory components of speech fluency, significant associations were followed up by Spearman’s partial correlations. For example, associations with speaking rate were followed up with partial correlation analyses that held constant the contribution of articulation rate.

## RESULTS

### Behavioral Correlations

**Figure 2.**
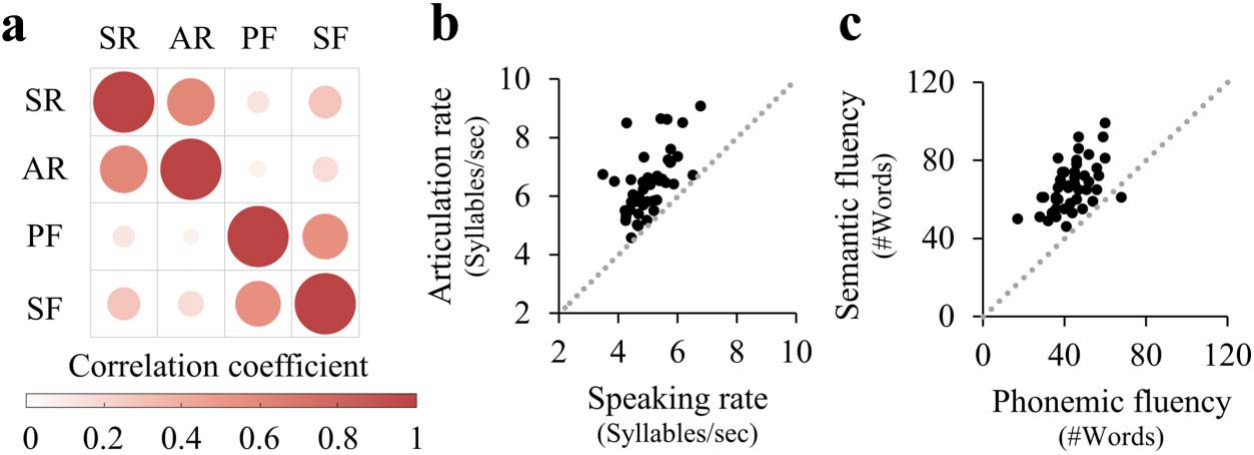
Behavioral dissociation between verbal fluency measures and speech rate measures. (A) Correlation matrix depicting two-tailed Spearman’s correlation coefficients between the four fluency measures calculated: speaking rate (SR), articulation rate (AR), phonemic fluency (PF), and semantic fluency (SF). The color saturation and size of the circles are proportional to the correlation coefficients. (B) and (C) depict the associations between speaking and articulation rate (B), and between phonemic and semantic fluency (C). Both correlations were significant after controlling the false discovery rate across all behavioral measures at *q* < 0.05. Dashed lines delineate *y* = *x*.

A correlation matrix of the four speech production measures (Figure 2A, Table S2) demonstrated significant correlations between the speaking and articulation rates (*r* = 0.599, *p* < 10^−4^), and between the semantic- and phonemic- fluency scores (*r* = 0.548, *p* < 10^−4^; both correlations were significant when controlling the FDR at *q* < 0.05). However, nonsignificant correlations were found between the verbal fluency measures and the speech rate measures (*p* > 0.06, uncorrected). Articulation rate was higher than speaking rate in all participants (Figure 2B; consistent with Amir, 2016). Semantic fluency scores were higher than phonemic fluency scores in 44/45 participants (Figure 2C; consistent with Kavé, 2005). Repeating this analysis with age standardized scores (Kavé & Knafo-Noam, 2015) generated a similar pattern of results (see Table S3).

### Tract Identification

The bilateral cerebellar peduncles and the bilateral FAT were successfully identified in all 45 participants (Figure 1). A qualitative examination of the tractograms (Figure 1A and B) and the individual tract profiles (Figure 1C and D) verified that the tracts were accurately segmented and provided consistently shaped profiles in all individuals (see Figure S3 for tract reconstruction in three representative participants).

### Speech Fluency Associations Within the Cerebellar Peduncles

To examine the relationships between speech fluency and the microstructural properties of the cerebellar peduncles in neurotypical adults, Spearman’s correlations were first calculated between the four measures of speech fluency (speaking rate, articulation rate, phonemic fluency, and semantic fluency) and mean tract diffusivities (tract FA and tract MD; see Materials and Methods) within each of the cerebellar peduncles. No significant correlations were detected between speech fluency and mean tract diffusivities in the cerebellar peduncles. Calculating the correlation for the age-standardized verbal fluency measures did not change these results. See Table S4 and Table S5 for a detailed list of correlation values.

**Figure 3.**
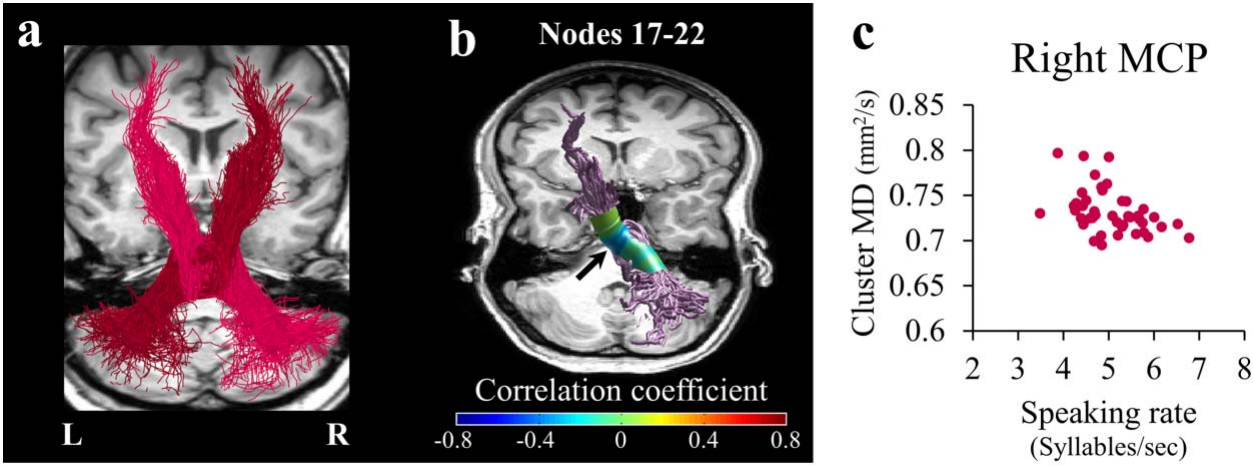
MD in the right MCP correlates with speaking rate. (A) The full trajectories of the right MCP (magenta) and the left MCP (burgundy) are shown in a representative subject (female, 27). (B) Two-tailed Spearman’s correlation coefficients are visualized in 30 nodes along the core of the right MCP. The black arrow denotes the location of the significant cluster of nodes (nodes 17–22, *p* < 0.05, familywise error corrected across 30 nodes). (C) A scatterplot showing the association between speaking rate and the averaged MD in the significant cluster of nodes within the right MCP. MCP = middle cerebellar peduncle; MD = mean diffusivity; L = left; R = right.

**Figure 4.**
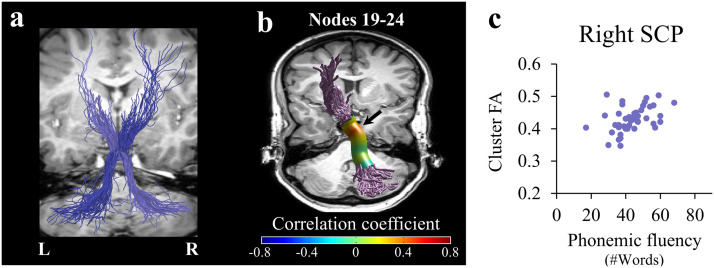
Fractional anisotropy in the right SCP correlates with phonemic fluency. (A) The full trajectories of the right SCP (light purple) and the left SCP (dark purple) are shown in a representative subject (female, 27). (B) Two-tailed Spearman’s correlation coefficients are visualized in 30 nodes across the core of the right SCP. The black arrow denotes the location of the significant cluster of nodes (nodes 19–24, *p* < 0.05, familywise error corrected across 30 nodes). (C) The scatterplot shows the association between phonemic fluency and mean FA in the significant cluster of nodes within the right SCP. SCP = superior cerebellar peduncle; FA = fractional anisotropy; L = left; R = right.

To achieve enhanced sensitivity for detecting localized brain-behavior correlations, we examined the relationships between speech fluency and local diffusivity values, node-by-node, along the trajectory of each cerebellar peduncle (see Materials and Methods). This analysis revealed a dissociation in the pattern of correlations within the cerebellar peduncles, such that MD within the right MCP was significantly correlated with speaking rate (*r* = −0.447, *p* < 0.05, nodes 17–22, FWE corrected across 30 nodes; Figure 3), whereas FA within the right SCP was significantly correlated with phonemic fluency (*r* = 0.431, *p* < 0.05, nodes 19–24, FWE corrected across 30 nodes; Figure 4). Note that both significant clusters were detected in the vicinity of the decussation. We address this point in the discussion.

Importantly, these correlations remained significant when calculating partial correlations, such that the correlation between the right MCP and speaking rate was not driven by phonemic fluency (*r* = −0.451, *p* = 0.002), and the correlation between the right SCP and phonemic fluency was not driven by speaking rate (*r* = 0.438, *p* = 0.003). Moreover, controlling for the contribution of articulation rate did not change these effects (Right MCP: *r* = −0.477, *p* = 0.001; Right SCP: *r* = 0.442, *p* = 0.003).

**Figure 5.**
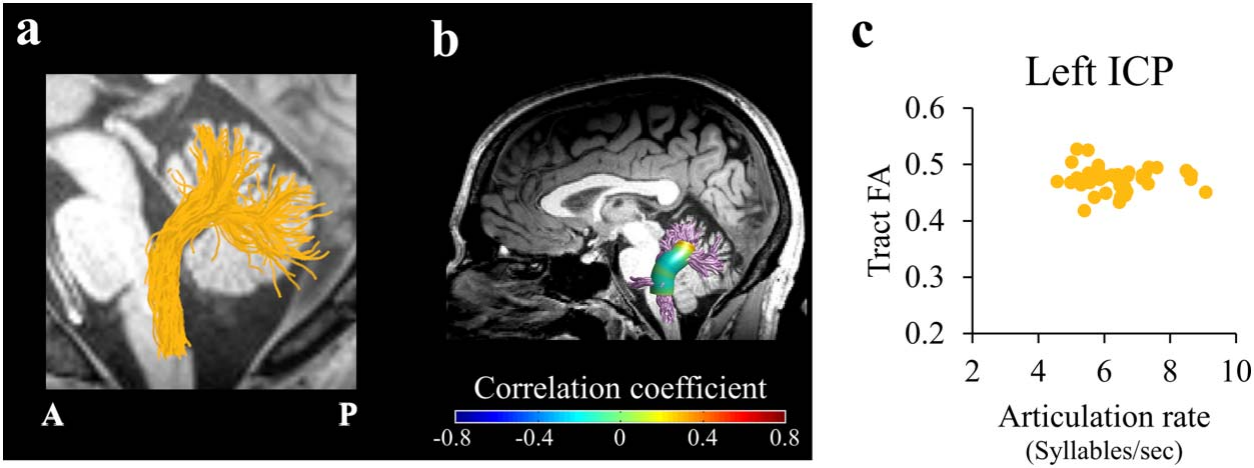
No correlation between the FA in left ICP and articulation rate. (A) The trajectory of the left ICP (yellow) is shown in a representative subject (female, 24). (B) Two-tailed Spearman’s correlation coefficients are visualized in 30 nodes across the core of the left ICP. (C) Articulation rate is plotted, for each participant, against tract FA (the mean FA across 30 nodes in the left ICP; see Materials and Methods). ICP = inferior cerebellar peduncle; FA = fractional anisotropy; A = anterior; P = posterior.

No additional significant correlations were found between the microstructural properties of the cerebellar peduncles and speech fluency measures (Table S4). Importantly, in line with a previous report (Jossinger et al., 2021), we did not find significant correlations between articulation rate and diffusivities within the left ICP of neurotypical adults (*p* > 0.5; Figure 5). Notice that, because there was no significant cluster of nodes in the ICP, the scatterplot in Figure 5C depicts tract FA (rather than cluster FA) against articulation rate.

### Speech Fluency Associations Within the Bilateral Frontal Aslant Tracts

**Figure 6.**
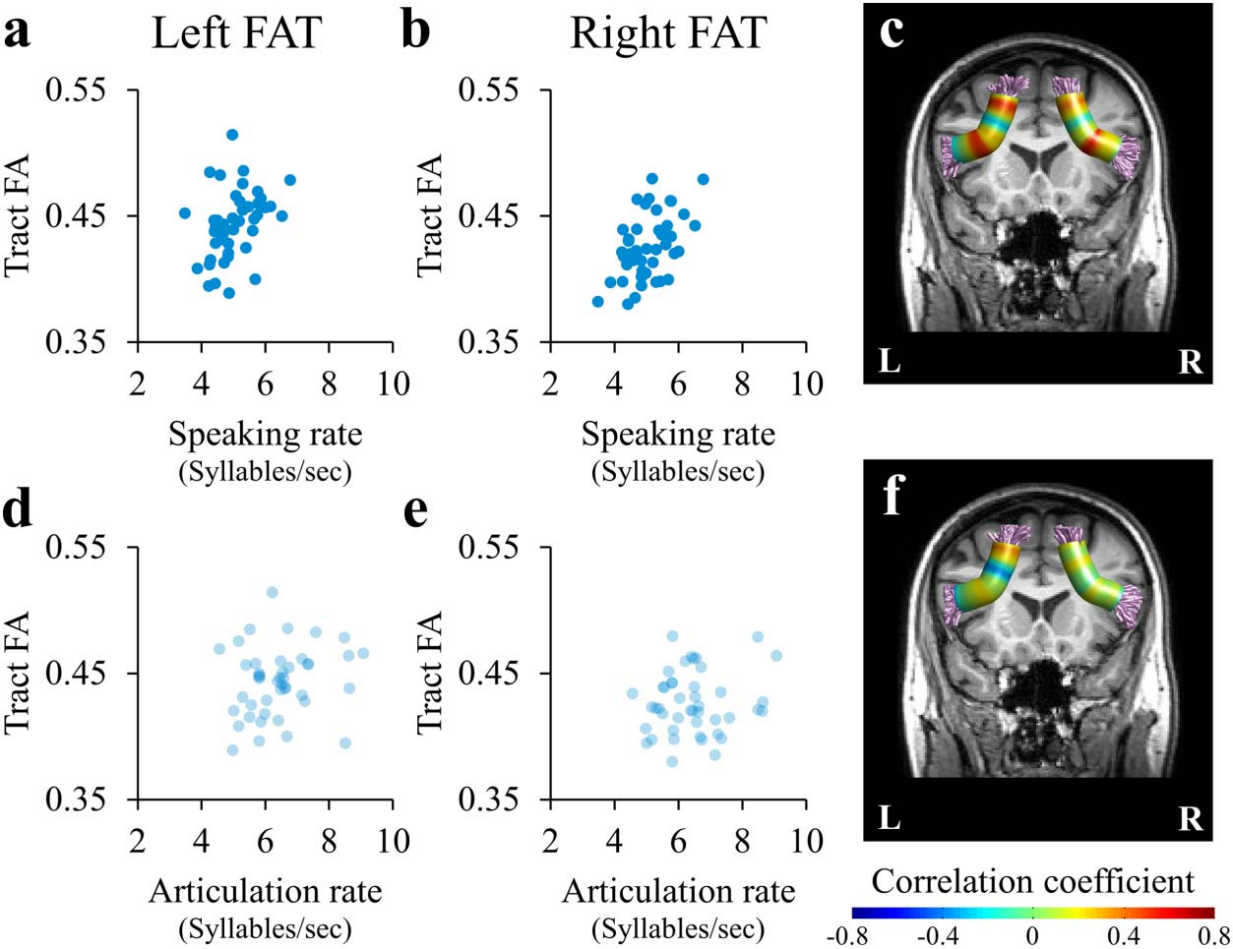
The bilateral FAT is associated with speaking rate, but not with articulation rate. Scatter plots show the association between the number of syllables per second and tract FA within the left FAT (A, D) and the right FAT (B, E). Tract FA in the left and right FAT significantly correlated with speaking rate (blue; A–B, *q* < 0.05, FDR corrected across all tracts), but not with articulation rate (light blue; D–E). For completeness, Spearman’s correlation coefficients between FA and speaking rate, and between FA and articulation rate are visualized in 30 nodes along the left and right FAT (C and F, respectively). The correlations along the tracts did not pass FWE correction for 30 nodes. FAT = frontal aslant tract; FA = fractional anisotropy; L = left; R = right.

To examine the relationships between speech fluency and the microstructural properties of the FAT in neurotypical adults, Spearman’s correlations were first calculated between the four measures of speech fluency (speaking rate, articulation rate, phonemic fluency, and semantic fluency) and tract diffusivities (tract FA and tract MD; see Materials and Methods) within the bilateral FAT (Table S4). This analysis revealed that tract diffusivities within the bilateral FAT were significantly correlated with speaking rate (Figure 6A–C). Specifically, speaking rate was positively correlated with tract FA within the left FAT (*r* = 0.459, *p* < 0.002; Figure 6A) and with tract FA within the right FAT (*r* = 0.466, *p* < 0.002; Figure 6B; both correlations were significant when controlling the FDR across eight tracts at *q* < 0.05). In both cases, neurotypical adults who speak faster have higher FA within their left and right FAT. In line with a previous report (Kronfeld-Duenias et al., 2016) no significant correlations were found between tract FA within the bilateral FAT and articulation rate (*p* > 0.1; Figure 6D–F). Moreover, the correlations between the bilateral FAT and speaking rate remained significant when calculating partial correlations, controlling for the effect of articulation rate (Left FAT: *r* = 0.465, *p* = 0.0014; right FAT: *r* = 0.488, *p* = 0.0007). Fisher’s *Z* test for dependent samples confirmed that the correlations between tract FA and speaking rate differed significantly from the correlations with articulation rate (left FAT: Fisher’s *Z* = 2.396, *p* < 0.009; right FAT: Fisher’s *Z* = 2.605, *p* < 0.006). See Table S4 and Table S5 for detailed correlation values between tract FA and tract MD values of the FAT and all speech fluency measures.

For completeness, we also calculated the correlations with speech fluency measures along the trajectory of the bilateral FAT (see Figure 6C for speaking rate and 6F for articulation rate). In line with tract FA results (Figure 6A and B), several nodes showed positive local correlations with speaking rate (Figure 6C). However, these associations did not survive the FWE correction. Significant negative correlation was detected between speaking rate and MD within the right FAT (nodes 10–24; *r* = −0.425, *p* < 0.05 FWE correction across 30 nodes; Figure S4). This correlation remained significant when calculating partial correlation, controlling for the effect of articulation rate (*r* = −0.325, *p* = 0.03). No other significant correlations were found between diffusivities within the bilateral FAT and speech fluency measures (Table S4).

## DISCUSSION

The goal of the current study was to evaluate the contribution of the CPs and the FAT to the fluent production of speech in neurotypical adults. Our findings demonstrate that the cerebellar peduncles dissociate between different speech fluency measures, such that the right MCP was associated with speaking rate while the right SCP was associated with phonemic fluency. These correlations were not driven by articulation rate. Further, we found that the bilateral FAT is also associated with speaking rate, but not with articulation rate. These findings shed light on the functional contributions of distinct subsystems involved in speech production. We discuss the findings concerning different aspects of speech fluency as they relate to the CPs and the FAT in the following sections.

### Dissociation Between Verbal Fluency and Speech Rate Measures

The production of fast and fluent speech requires interaction between several processes, including high-level linguistic components, such as lexical access and phonological encoding, and motor components which are crucial for articulatory control. In the current study we assessed these different components by using different types of tasks that require overt production of speech: verbal fluency tasks and an unstructured interview. Our data show a behavioral dissociation between verbal fluency measures and speech rate measures (Figure 2). Moreover, we found that specific fluency measures mapped onto different white matter tracts. Together, these findings suggest that different fluency measures may reflect different aspects of speech production.

#### Task differences

The tasks we used to evaluate speech fluency differ in several aspects. First, an unstructured interview is a relatively naturalistic setting of speech production, while verbal fluency tasks measure speech production in a more artificial way. Second, producing a free narrative involves conceptualizing the message and projecting a syntactic structure, while a verbal fluency task elicits a list of words, mostly unconnected nouns, based on a predetermined criterion. Third, each task may involve different cognitive demands beyond speech production. For example, verbal fluency tasks require the use of executive functions such as response selection, the inhibition of irrelevant responses, and more (Friedman et al., 1998; Kramer et al., 2014). Spontaneously speaking during an unstructured interview, on the other hand, may be effected by the social and emotional state of the speaker (Amir, 2016). The extent to which each of the two speech fluency tasks used in this study relies on other cognitive functions, which are not speech-related, remains to be studied directly in future studies.

#### What does speaking rate actually measure?

Despite the considerable correlation between articulation rate and speaking rate (Figure 2B), the two metrics are thought to represent different aspects of speech production. Speaking rate, which is calculated over continuous segments of speech, including disfluencies of various sources, is considered a global measure of verbal output and language proficiency (Costello & Ingham, 1984). Articulation rate, in contrast, is calculated over the same speech samples but after excluding disfluent segments, thus thought to reduce linguistic effects and to represent articulatory motor control (Walker et al., 1992).

**Figure 7.**
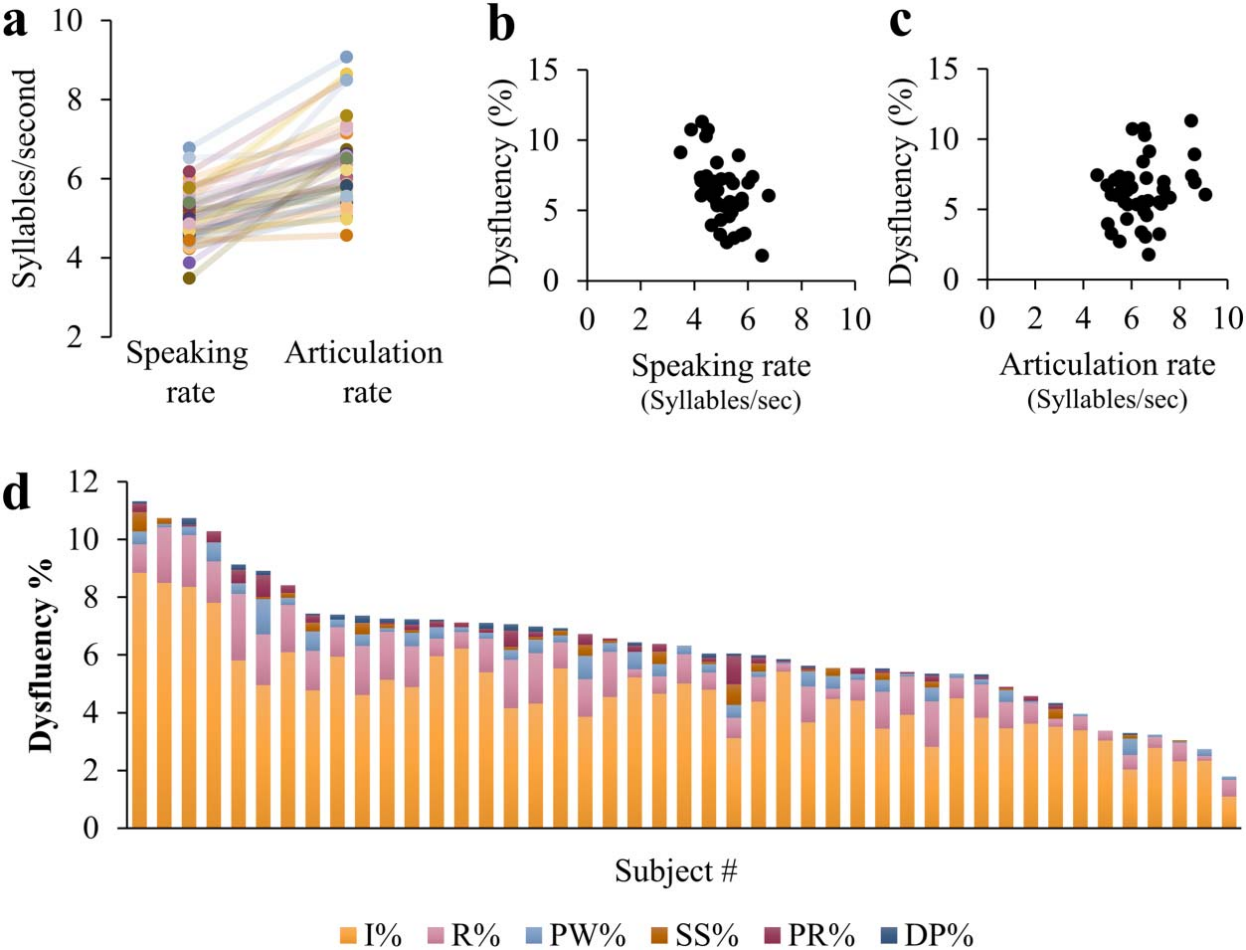
Analysis of individual speaking rate. (A) Individual speaking rates and articulation rates. Each line represents a single participant. In all participants, speaking rate is slower compared to articulation rate. (B–C) Speaking rate, but not articulation rate, is significantly associated with the percent of dysfluencies (*r* = −0.51, *p* = 0.0003 compared with *r* = 0.09, *p* = 0.56), such that slower speakers demonstrate more dysfluencies while they speak. (D) Distribution of dysfluencies by type. In our sample of neurotypical speakers, most dysfluencies included interjections (I%; e.g., “um”) and revisions (R%; e.g., “so I was, we were…”). Other dysfluencies that occurred at much lower rates were part-word repetitions (PW%; e.g., “In, in..”), stuttered syllables (SS%; e.g., “The, the..”), phrase repetitions (PR%; e.g., “because the flight was … the flight was”), and dysrhythmic phonation (DP%; e.g., “looooooves”).

To better understand the sources of the differences between speaking and articulation rates, we conducted a more elaborate analysis of the individual speech segments, as described in Figure 7. As expected, in all participants, speaking rate is slower compared to articulation rate (Figure 7A). This difference may reflect naturally occurring disfluencies, which are included in the calculation of speaking rate, not articulation rate. In line with this view, speaking rate, but not articulation rate, is negatively correlated with the frequency of dysfluencies, such that slower speakers demonstrate more dysfluencies (compare Figure 7B with 7C). In our sample of neurotypical adults, the vast majority of dysfluencies included interjections (e.g., “um”) and revisions (e.g., “so I was, we were …”; see Figure 7D). Such naturally occurring dysfluencies are commonly observed in neurotypical speakers and may reflect difficulties in formulating the message at different levels, for example, the conceptual, syntactic, or pragmatic levels.

### Cerebellar Internal Models of Speech and Language

The cerebellum has long been viewed as a motor control structure. Over the past two decades, however, evidence points to a more general role for the cerebellum in the modulation of higher-level cognitive processes (Schmahmann, 2010). Our findings support this view by showing that the white matter pathways that connect the cerebellum to the cerebrum are important for the modulation of both speaking rate and phonemic fluency. Importantly, these correlations remain significant after we partial out the effect of articulation rate, a proxy for motor control. Therefore, the selective association patterns highlight the unique contribution of the cerebellum to linguistic, rather than motor components of speech, in neurotypical adults.

#### Cerebellar internal models

Studies focusing on the cerebellar computations postulate that it encodes “internal models,” corresponding to the neural representations that we generate for the external world (Ito, 2008; Kawato, 1999; Shadmehr & Krakauer, 2008; Wolpert et al., 1998). This concept developed from contemporary theories of motor control, arguing that cerebellar internal models capture the causal relationships between a voluntary movement and its consequence. Internal models are used to predict the outcome of an action before the arrival of the actual sensory feedback from the environment. This mechanism is often used to explain how fast and coordinated movements can be carried out accurately, regardless of the slow nature of sensory feedback loops (Shadmehr & Krakauer, 2008; Wolpert et al., 1998). Indeed, current models of speech production argue that cerebellar internal models are used to transform motor-to-sensory information which is crucial for articulation, thus enabling fast and fluent production of speech (Guenther, 2006; Hickok, 2012).

#### Internal models of language in the cerebellum

The accumulative evidence implicating the cerebellum in high-level cognitive functions raises the possibility that cerebellar internal models are utilized to simulate mental representations, similarly to the manner in which they operate on motor behaviors (Ito, 2008). With respect to language processing, cerebellar internal models are hypothesized to store memories of sequential linguistic events, such as phonological, semantic, and syntactic sequences, which are used to predict upcoming linguistic events during speech comprehension (Argyropoulos, 2016). By showing an association between the cerebro-cerebellar pathways and speech production, we may speculate not only that the cerebellum functions as an internal model during speech comprehension but also that it may contribute to prediction of linguistic elements during speech production.

### Cerebro-Cerebellar Pathways Mediate Speech Fluency in Neurotypical Adults

The hypotheses regarding the involvement of the cerebellum in cognition and language are largely based on the anatomical connections between the cerebellum and the cerebral cortex. In the late 1900s it was suggested that in humans, the increased size of the cerebellum and cerebral cortex led to the formation of new cerebro-cerebellar pathways that may contribute to high-level functions such as language (Leiner, 2010; Leiner et al., 1986). Connections between the cerebellum and the prefrontal cortex were indeed traced in both monkeys (Middleton & Strick, 1994) and humans (Palesi et al., 2015, 2017). Recently, the cerebro-cerebellar pathways were implicated in various reading tasks that require overt production of speech (Bruckert et al., 2020; Travis et al., 2015). This is the first study, however, that shows a direct link between the microstructural properties of the cerebro-cerebellar pathways and speech fluency that goes beyond articulation, providing an anatomical and functional support to the hypotheses made in the 1980s (Leiner et al., 1986).

#### The cerebro-cerebellar pathways dissociate between speech fluency measures

Our results point to distinct patterns of association between speech fluency measures and the microstructural properties of the cerebro-cerebellar pathways. Specifically, we found that the right SCP is associated with phonemic fluency (but not with speaking rate; see Figure 4 and accompanying text), while the right MCP is associated with speaking rate (but not with phonemic fluency; see Figure 3 and accompanying text). The difference in the functionalities associated with each peduncle is in line with the difference in the type of signals conveyed by each: The SCP is mainly an output pathway, transmitting signals from the cerebellar deep nuclei into the contralateral cerebral hemisphere, while the MCP is mainly an input pathway, feeding signals from the cerebral cortex into the contralateral cerebellar hemisphere. We may speculate that the involvement of the right SCP in phonemic fluency reflects the retrieval of phonological sequences from the cerebellar internal model, while the involvement of the right MCP in spontaneous speaking rate reflects the evaluation of the linguistic content against its internal representation. Future neurophysiological investigations will be essential to directly test this interpretation.

#### Functional lateralization within the cerebellum

Our findings demonstrate a functional lateralization within the cerebellum, by associating linguistic aspects of speech production with microstructural properties of the right SCP and right MCP. The concept of the “lateralized linguistic cerebellum,” implicating the right cerebellar hemisphere in linguistic operations (Marien et al., 2001), has been well established in previous studies. For example, tasks that require lexical access and word retrieval were shown to activate the inferior lateral part of the right cerebellar hemisphere in neurotypical adults (Petersen et al., 1988, 1989). Further, patients with damage to the right cerebellar hemisphere were shown to have a specific deficit in tasks that require semantic retrieval (Fiez et al., 1992) or grammatical production (Silveri et al., 1994; Zettin et al., 1997). The right cerebellar hemisphere is reciprocally connected to the left cerebral hemisphere via the right SCP and the right MCP. Our findings provide an independent support in healthy adults for the involvement of the right cerebellum in speech and language.

#### No significant correlations between the CPS and articulation rate in neurotypical adults

Articulation rate was not associated with the microstructural properties in any of the CPs among our sample of neurotypical adults (Table S4). Of particular relevance is the nonsignificant association between articulation rate and diffusivities within the left ICP (Figure 5), in agreement with our previous findings in an independent, smaller sample of neurotypical adults (Jossinger et al., 2021). In that study, articulation rate was associated with FA along the left ICP only in a group of adults with persistent developmental stuttering and not in age-matched fluent speakers (see figure 6 in Jossinger et al., 2021). The ICP, transmitting sensory feedback signals from the periphery to the cerebellum, was repeatedly implicated in encoding motor errors during various motor tasks, including reaching movements (Shadmehr, 2017) and locomotion (Jossinger et al., 2020), which are crucial for the formation and updating of speech-related internal models (Tourville & Guenther, 2011). It was suggested that adults who stutter rely too heavily on sensory feedback while they speak due to a noisy or insufficient internal model (Hickok et al., 2011; Max et al., 2004). In neurotypical adults, on the other hand, a well-functioning internal model may be sufficient for producing fast and fluent speech, thus diminishing the speakers’ dependency on sensory feedback in order to produce adequate articulatory output.

An alternative explanation for the lack of correlation between articulation rate and the microstructural properties of the ICP may rest in the type of task used to evaluate articulation rate. In clinical populations, diagnosed with various speech disorders, articulation rate is typically calculated over audio recordings of an unstructured interview. In neurotypical speakers, however, natural speaking may be insufficient to challenge the articulatory system in order to reveal its underlying neural mechanisms. To cope with this limitation, future studies could use the diadochokinetic rate, a task designed to stress the articulatory system by measuring how quickly a person can produce a series of alternating sounds.

### The Involvement of the Frontal Aslant Tract in Speech Fluency

The FAT connects two cerebral areas important for speech production: the posterior IFG (also known as Broca’s area) with the pre-SMA and SMA (Catani et al., 2013). Accumulative evidence from the last decade, mostly from clinical data, suggests that the FAT is a key pathway for speech fluency, as detailed below. The current study supplies further anatomical evidence for the involvement of the FAT in the fluent production of speech.

#### The FAT mediates speaking rate in neurotypical adults

Our results show a positive correlation between diffusivity of the bilateral FAT and speaking rate in neurotypical adults (Figure 6A and B; Figure S4). Specifically, we found that adults who speak faster have higher mean FA within their left and right FAT, and lower MD within their right FAT. The involvement of the FAT in speaking was previously demonstrated in patients. Electrical stimulation of the FAT during neural surgery was shown to elicit speech arrest (Fujii et al., 2015; Kinoshita et al., 2015; Vassal et al., 2014). In patients with primary-progressive aphasia and in patients with multiple sclerosis, the microstructural properties of the FAT were correlated with measures of speech rate and verbal fluency (Blecher et al., 2019; Catani et al., 2013; Li et al., 2017). To the best of our knowledge, this is the first study that shows a direct involvement of the bilateral FAT in speech production among healthy subjects.

#### No significant correlation between the FAT and articulation rate in neurotypical adults

Although speaking rate and articulation rate are highly correlated (Figure 2B), the bilateral FAT was associated with speaking rate but not with articulation rate, and the difference between these correlation coefficients was significant. These findings are in line with previous results showing a significant association between the left FAT and articulation rate only in adults with persistent developmental stuttering, not in neurotypical adults (Kronfeld-Duenias et al., 2016). A recent study, investigating the same sample of neurotypical adults reported here, found that the bilateral FAT was also implicated in a morphological task that requires overt speech, beyond the contribution of verbal fluency scores (Yablonski et al., 2021). Taken together, these findings suggest that, in neurotypical adults, the FAT contributes to higher-level aspects of language production, rather than to articulatory motor control per se.

### Interpreting the Results in Terms of Tissue Properties

Both FA and MD are modulated by multiple biological factors, such as axonal density, directional coherence, axonal diameter, and myelin content, which may influence FA and MD in opposite directions (Assaf & Pasternak, 2008; Beaulieu, 2002; Jones et al., 2013). For example, tight axonal packing or elevated myelination which lead to better neuronal communication, would manifest in elevated FA but reduced MD in the same voxel. On the other hand, wider axonal diameter, promoting efficient conduction (Liewald et al., 2014), would result in reduced FA and elevated MD (Barazany et al., 2009; Horowitz et al., 2015). Broadly speaking, however, FA and MD are not negatively correlated across different pathways and may therefore reflect different components of the underlying tissue (De Santis et al., 2014; Uddin et al., 2019).

In our data, better phonemic fluency and faster speaking rate are associated with higher FA values in the right SCP and the bilateral FAT, respectively. Such positive correlations may be mediated by axonal packing or myelination: elevated axonal packing (or myelin content) would lead to both higher FA and better conductance, hence better performance. In addition to the positive correlations observed with FA metrics, negative correlations between MD and speaking rate were detected in the right MCP and the right FAT. Following the same line of reasoning developed earlier, one scenario that could lead to this pattern of results suggests that tightly packed axons, which are linked to better performance, also restrict the overall diffusivity in a voxel, thus causing a reduction in the local MD values in association with improved performance.

In sum, the directions of the associations we found are internally consistent, and are compatible with some explanations in terms of tissue properties and not with others. These explanations may be further tested with advanced quantitative MRI measures that are sensitive to the different biological factors comprising the tissue (Assaf & Basser, 2005; Assaf et al., 2008; Berman et al., 2019; Filo et al., 2019; Mezer et al., 2013).

### Limitations

The current study takes a tract-based approach, in which we test a small number of predefined hypotheses using individualized tractography methods in native space. This approach limits the number of hypotheses tested, as adding more tracts to the analysis would increase the chances of false positive results. Here, we focused on the bilateral CPs and FAT that were previously associated with different aspects of speech fluency in clinical populations (Jossinger et al., 2021; Kronfeld-Duenias et al., 2016). However, we do not rule out the possibility that other white matter tracts may also contribute to various aspects of speech production. Specifically, the basal ganglia were previously shown to be implicated in speech fluency, in both clinical and neurotypical populations (Booth et al., 2007; Chang & Guenther, 2020; Zenon & Olivier, 2014). Future studies will be needed to test whether basal ganglia connections underpin speech fluency in neurotypical speakers.

A second limitation concerns the location of the correlations along the right SCP and right MCP, which were found in the vicinity of the decussation. At the point of decussation, voxels are occupied by both right and left tracts, making it difficult to attribute the findings to either the left or the right tract. Considering that voxels in the decussation of the CPs contain two fiber orientations (as shown by Jeurissen et al., 2013), such voxels likely contain primarily the two crossing CPs (e.g., left and right SCP), uncontaminated by other tracts. Nonetheless, crossing fibers are known to affect (typically reduce) FA values (Figure 1C–D). The FA drop near the decussation is not sufficient, however, to generate a significant correlation, as the significant results with FA at the vicinity of decussation were specific to the right SCP and were not shown in other pathways that contain decussating fibers. The effect of crossing fibers on MD in our data is less dramatic: MD profiles (particularly in the MCP and ICP; see Figure S4C–F) are generally flatter compared to FA profiles (Figure 1C and D). Therefore, the fact that we find a significant correlation with MD in the right MCP suggests that this effect is not driven only by crossing fibers.

Lastly, the current study, as any other study that identifies correlations in a sample of adults, cannot be conclusive about the direction of causality that underlies the effects. Future developmental and longitudinal studies will help in determining whether the variability in the microstructure of the CPs and the FAT derives the variability in speech fluency seen in adulthood, or perhaps the differences in microstructure arise as a result of other cognitive and motor tasks involved in speech fluency.

## CONCLUSIONS

Our findings support the involvement of the cerebellum in aspects of speech production that go beyond articulatory control, such as lexical access, pragmatic or syntactic generation. Using complex modeling and probabilistic tracking enabled us to follow the trajectory of the cerebro-cerebellar pathways as they decussate and to detect novel associations with speech fluency in these pathways. By evaluating multiple measures of speech fluency, our study makes an important contribution to the understanding of the neural basis of speech production in neurotypical adults.

## ACKNOWLEDGMENTS

This study was conducted as part of Sivan Jossinger’s doctoral dissertation, carried out under the supervision of Professor Michal Ben-Shachar at the Gonda Multidisciplinary Brain Research Center, Bar-Ilan University. We thank Yaniv Assaf, Daniel Barazany, and the team at the Strauss Center for Computational Neuroimaging for their assistance in protocol setup and MRI setting. We are thankful to an anonymous NOL reviewer for their idea regarding diadochokinetic rate in future studies.

## FUNDING INFORMATION

Michal Ben-Shachar, Israel Science Foundation (https://dx.doi.org/10.13039/501100003977), Award ID: 1083/17.

## AUTHOR CONTRIBUTIONS

**Sivan Jossinger**: Conceptualization: Equal; Formal analysis: Lead; Investigation: Equal; Visualization: Lead; Writing – original draft: Equal; Writing – review & editing: Equal. **Maya Yablonski**: Data curation: Lead; Investigation: Equal; Writing – review & editing: Equal. **Ofer Amir**: Funding acquisition: Equal; Supervision: Equal; Writing – review & editing: Equal. **Michal Ben-Shachar**: Conceptualization: Equal; Funding acquisition: Equal; Investigation: Equal; Supervision: Lead; Writing – review & editing: Equal.

## Supplementary Material



## TECHNICAL TERMS

Phonemic fluency: Number of words beginning with a predefined letter, produced in a limited time frame.
Semantic fluency: Number of words belonging to a predefined category, produced in a limited time frame.
Speaking rate: The number of syllables produced per second calculated over spontaneous speech samples.
Articulation rate: The number of syllables produced per second calculated only over fluent segments within spontaneous speech.
Diffusion MRI (dMRI): An MRI-based imaging method which measures the speed and directionality of water diffusion in biological tissues.
Tensor modeling: A simple computational model describing speed and directionality of diffusion as an ellipsoid fit to dMRI data in each voxel.
Constrained spherical deconvolution (CSD): A complex computational model of dMRI data that estimates the orientation of multiple fibers within each voxel.
Fractional anisotropy: A measure of the degree of directional diffusion based on the tensor model.
Mean diffusivity (MD): A measure of the average speed of diffusion based on the tensor model.
Tractogram: A 3D representation of virtual fibers generated from dMRI data.
