# Supplementary material for: The Contributions of the Cerebellar Peduncles and the Frontal Aslant Tract in Mediating Speech Fluency
Source: Neurobiol Lang (Camb). 2024 Aug 15;5(3):676–700. doi: 10.1162/nol_a_00098 (PMC11338307; doi:10.1162/nol_a_00098)
Supplement: Supplementary file 1 [file nol-5-3-676-s001.pdf]

The contributions of the cerebellar peduncles and the frontal aslant tract in mediating speech fluency

Supplementary material

Figure S1. Segmentation of the cerebellar peduncles. a) ROIs drawn on the MNI152 template. b) ROIs transformed to the native space of an individual participant (female, 24). c) Individual whole brain tractogram generated with probabilistic tractography (for visualization purposes, only a random subset of 1500 streamlines is shown). d) The resulting CPs segmented by intersecting the whole brain tractogram (c) with the waypoint ROIs (b). SCP- purple; MCP- magenta; ICP- yellow. Abbreviations: SCP – superior cerebellar peduncle; MCP – middle cerebellar peduncle; ICP – inferior cerebellar peduncle; ROI – region of interest.

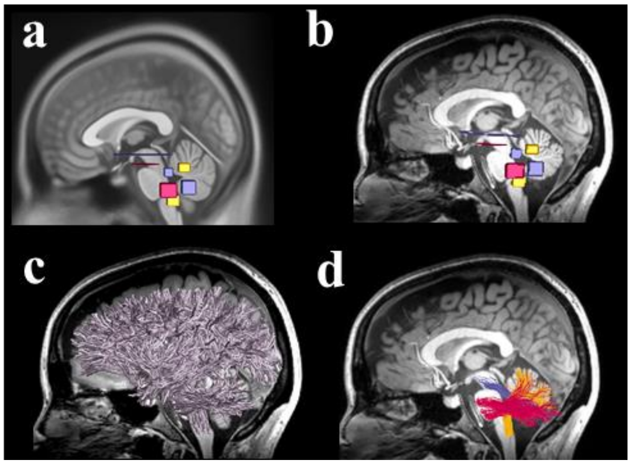

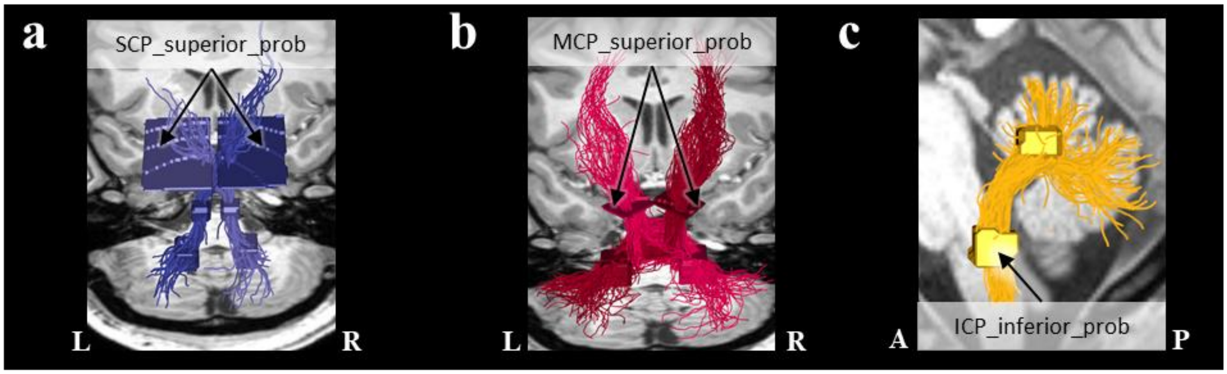

Figure S2. Cerebellar peduncles with their corresponding ROIs. The bilateral SCP (a; left SCP- dark purple, right SCP- light purple), bilateral MCP (b; left MCP- burgundy, right MCP- magenta) and the left ICP (c; yellow) are shown with their corresponding ROIs in a single participant (female, 24), overlaid on a T1 image of the same individual. Black arrows denote the newly defined ROIs. Abbreviations: SCP – superior cerebellar peduncle; MCP – middle cerebellar peduncle; ICP – inferior cerebellar peduncle; ROI – region of interest; L – left; R – right; A – anterior; P – posterior.

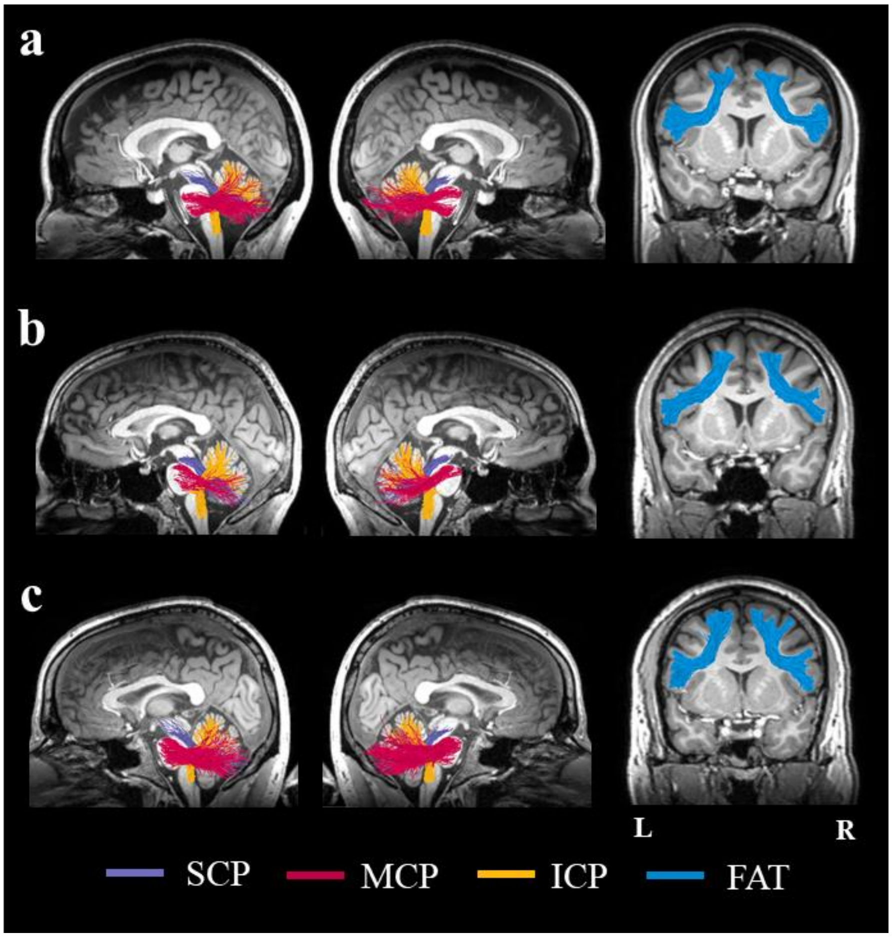

Figure S3. Tracts of interest in 3 representative participants. The left and right tracts of interest are demonstrated in three representative subjects: (a) female, 24; (b) male, 34; (c) male, 30. Tracts are overlaid on a midsagittal T1 image of each participant. The cerebellar tracts identified are the superior cerebellar peduncle (SCP; purple), middle cerebellar peduncle (MCP; magenta), and the inferior cerebellar peduncle (ICP; yellow). Also identified are the bilateral frontal aslant tracts (FAT; blue). Abbreviations: SCP – superior cerebellar peduncle; MCP – middle cerebellar peduncle; ICP – inferior cerebellar peduncle; FAT – frontal aslant tract; L – left; R - right.

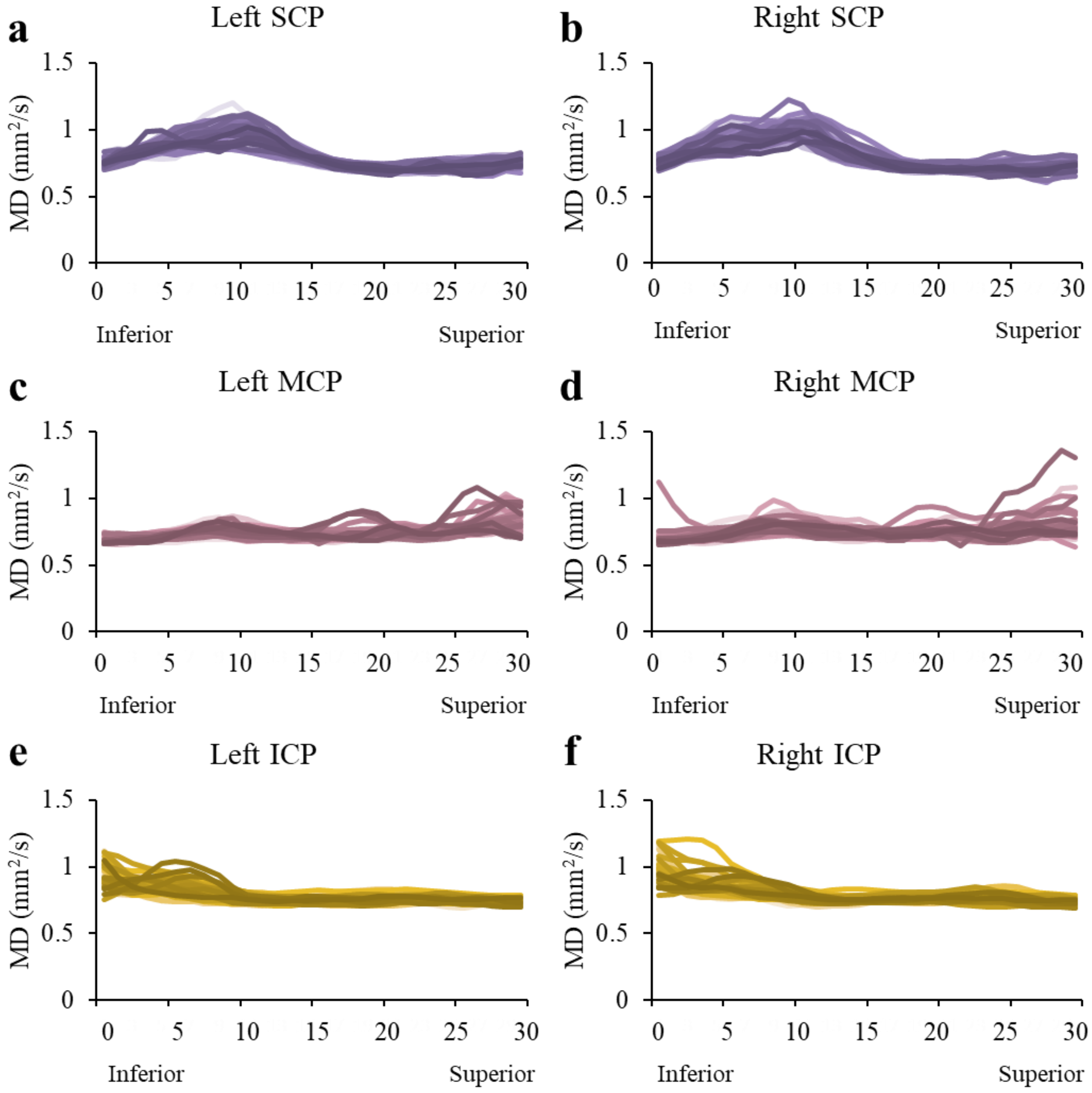

Figure S4. Tract mean diffusivity (MD) profiles of the bilateral cerebellar peduncles (CPs). Individual tract profiles show MD values at 30 equidistant nodes along the core of the bilateral SCP (a-b; purple), MCP (c-d; magenta), and ICP (e-f; yellow). Each subject is represented by a single line (N=45).

Abbreviations: SCP – superior cerebellar peduncle; MCP – middle cerebellar peduncle; ICP – inferior cerebellar peduncle; MD – mean diffusivity.

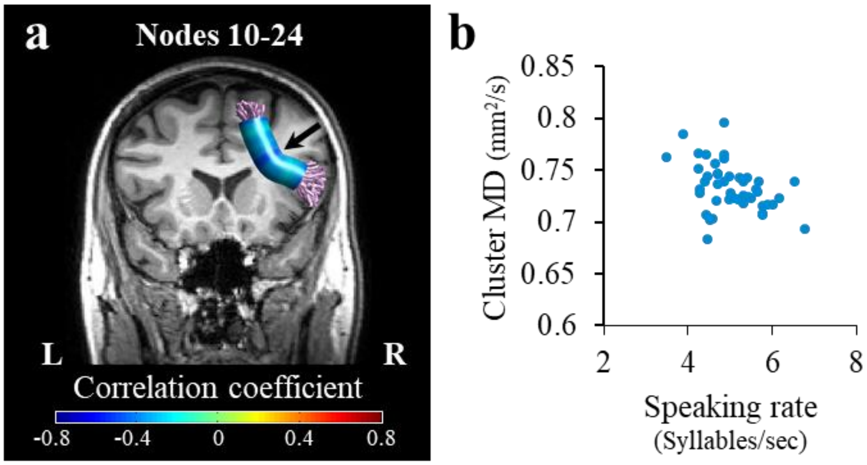

Figure S5. Mean diffusivity in the right frontal aslant tract correlates with speaking rate. (a) Two-tailed Spearman's correlation coefficients are visualized in 30 nodes along the core of the right FAT. The black arrow denotes the center of the significant cluster of nodes (nodes 10-24, $p<0.05$, family-wise error corrected across 30 nodes). (b) A scatter plot showing the association between speaking rate and the average MD in the significant cluster of nodes within the right FAT. Abbreviations: FAT – frontal aslant tract; MD – mean diffusivity; L – left; R – right.

.

Table S1. MNI coordinates of ROIs used to segment the cerebellar peduncles[a-f].

ROI	X [medial, lateral]	Y [anterior, posterior]	Z [superior, inferior]
SCP_L_inferior_prob	[-10, -18]	[-50, -60]	[-32, -42]
SCP_L_inter_prob	[-4, -10]	[-36, -42]	[-28, -22]
SCP_L_superior_prob	[-2, -32]	[7, -40]	[-10, -10]
SCP_R_inferior_prob	[10, 18]	[-50, -60]	[-32, -42]
SCP_R_inter_prob	[4, 10]	[-36, -42]	[-28, -22]
SCP_R_superior_prob	[2, 32]	[7, -40]	[-10, -10]
MCP_L_inferior_prob	[-12, -22]	[-32, -44]	[-34, -44]
MCP_L_superior_prob	[0, -22]	[-8, -30]	[-18, -18]
MCP_R_inferior_prob	[12, 22]	[-32, -44]	[-34, -44]
MCP_R_superior_prob	[0, 22]	[-8, -30]	[-18, -18]
ICP_L_inferior_prob	[-3, -13]	[-38, -47]	[-44, -52]
ICP_L_superior_prob	[-3, -13]	[-47, -57]	[-17, -25]
ICP_R_inferior_prob	[3, 13]	[-38, -47]	[-44, -52]
ICP_R_superior_prob	[3, 13]	[-47, -57]	[-17, -25]

[a] Left SCP was segmented using the SCP_L_inferior_prob and the SCP_R_superior_prob as waypoint ROIs; SCP_L_inter_prob was added as an AND-ROI ; SCP_L_superior_prob was used as a NOT-ROI.

[b] Right SCP was segmented using the SCP_R_inferior_prob and the SCP_L_superior_prob as waypoint ROIs; SCP_R_inter_prob was added as an AND-ROI ; SCP_R_superior_prob was used as a NOT-ROI.

[c] Left MCP was segmented using MCP_L_inferior_prob and MCP_R_superior_prob as waypoint ROIs; SCP_L_inter_prob was used as a NOT-ROI.

[d] Right MCP was segmented using MCP_R_inferior_prob and MCP_L_superior_prob as waypoint ROIs; SCP_R_inter_prob was used as a NOT-ROI.

[e] Left ICP was segmented using the ICP_L_inferior_prob and the ICP_L_superior_prob as waypoint ROIs.

[f] Right ICP was segmented using the ICP_R_inferior_prob and the ICP_R_superior_prob as waypoint ROIs.

Table S2. Correlations between speech rate measures and raw verbal fluency scores[a].

	Speaking rate	Articulation rate	Phonemic fluency
Articulation rate	**0.599***		
Phonemic fluency	0.126	0.067	
Semantic fluency	0.282	0.168	**0.548***

[a] The table depicts two-tailed Spearman's correlation coefficients between the behavioral (N=45).

* *Uncorrected* $p<10^{-4}$, significant after controlling the FDR across all behavioral measures at q < 0.05.

Table S3. Correlations between speech rate measures and standardized verbal fluency scores[a].

	Speaking rate	Articulation rate	Phonemic fluency
Articulation rate	**0.599***		
Phonemic fluency	0.111	0.069	
Semantic fluency	0.303[§]	0.170	**0.522***

[a] The table depicts two-tailed Spearman's correlation coefficients between the behavioral measures (N=45).

* *Uncorrected* $p<10^{-4}$, significant after controlling the FDR across all behavioral measures at q < 0.05.

[§] *Uncorrected* $p<0.05$, non-significant when controlling the FDR at q < 0.05.

Table S4. Correlations between fluency measures and mean-tract diffusivities[a].

	Speaking rate		Articulation rate		Phonemic fluency		Semantic fluency	
	r	*p*	*r*	*p*	*r*	*p*	*r*	*p*
Tract-FA								
left SCP	0.152	0.319	-0.031	0.840	0.150	0.325	0.139	0.359
right SCP	0.029	0.848	-0.061	0.690	0.180	0.236	0.204	0.178
left MCP	0.119	0.434	-0.094	0.540	0.072	0.640	0.315	**0.034**§
right MCP	0.126	0.409	-0.109	0.474	0.041	0.792	0.100	0.513
left ICP	-0.039	0.799	-0.029	0.849	-0.002	0.990	-0.031	0.835
right ICP	0.087	0.568	0.028	0.854	0.094	0.540	-0.033	0.828
left FAT	0.459	**0.002***	0.152	0.319	-0.031	0.843	0.299	**0.045**§
right FAT	0.466	**0.001***	0.131	0.390	0.041	0.789	0.291	0.052
Tract-MD								
left SCP	-0.206	0.174	-0.185	0.223	-0.146	0.338	-0.044	0.774
right SCP	-0.074	0.629	-0.234	0.121	-0.195	0.199	-0.087	0.567
left MCP	-0.184	0.226	-0.197	0.194	-0.216	0.154	-0.082	0.592
right MCP	-0.151	0.322	-0.185	0.222	-0.375	**0.011**§	-0.147	0.333
left ICP	-0.131	0.389	-0.101	0.508	0.100	0.512	0.038	0.801
right ICP	-0.097	0.525	-0.031	0.840	-0.052	0.733	-0.022	0.881
left FAT	-0.345	**0.021**§	-0.137	0.370	-0.098	0.523	-0.272	0.070
right FAT	-0.413	**0.005***	-0.200	0.188	-0.191	0.210	-0.367	**0.013**§

[a] The table depicts two-tailed Spearman's correlation coefficients and p-values between the behavioral measures and mean-tract diffusivities (N=45).

* *Uncorrected p≤0.005*, significant when controlling the FDR across all behavioral measures at q<0.05.

§ *Uncorrected p<0.05*, non-significant when controlling the FDR at q < 0.05.

Table S5. Correlations between standardized verbal fluency measures and mean-tract diffusivities[a].

	Phonemic fluency Z scores		Semantic fluency Z scores	
	r	*p*	*r*	*p*
Tract-FA				
left SCP	0.118	0.439	0.132	0.385
right SCP	0.143	0.348	0.200	0.186
left MCP	0.071	0.642	0.324	0.029
right MCP	0.024	0.874	0.118	0.436
left ICP	0.001	0.992	-0.052	0.734
right ICP	0.0801	0.601	-0.047	0.75
left FAT	-0.033	0.825	0.295	**0.048**[§]
right FAT	0.017	0.910	0.313	**0.036**[§]
Tract-MD				
left SCP	-0.150	0.322	-0.056	0.711
right SCP	-0.210	0.164	-0.058	0.703
left MCP	-0.207	0.172	-0.112	0.461
right MCP	-0.374	**0.011**[§]	-0.179	0.239
left ICP	0.098	0.520	0.032	0.830
right ICP	-0.077	0.613	-0.035	0.817
left FAT	-0.090	0.555	-0.286	0.056
right FAT	-0.179	0.238	-0.384	**0.009**[§]

[a] The table depicts two-tailed Spearman's correlation coefficients and p-values between the standardized verbal fluency scores and mean-tract (N=45).

[§] *Uncorrected p<0.05*, non-significant when controlling the FDR at q < 0.05.
